# On the Genesis of Artifacts in Neutron Transmission Imaging of Hydrogenous Steel Specimens

**DOI:** 10.3390/jimaging6040022

**Published:** 2020-04-09

**Authors:** Beate Pfretzschner, Thomas Schaupp, Andreas Hannemann, Michael Schulz, Axel Griesche

**Affiliations:** 1 Bundesanstalt für Materialforschung und –prüfung (BAM), Unter den Eichen 87, 12205 Berlin, Germany; beate.pfretzschner@bam.de (B.P.); thomas.schaupp@bam.de (T.S.); andreas.hannemann@bam.de (A.H.); 2 Heinz Maier-Leibnitz Zentrum (MLZ), Technische Universität München, Lichtenbergstr. 1, 85748 Garching, Germany; Michael.Schulz@frm2.tum.de

**Keywords:** neutron imaging, hydrogen, supermartensitic steel, backlight, scattering, refraction

## Abstract

Hydrogen-charged supermartensitic steel samples were used to systematically investigate imaging artifacts in neutron radiography. Cadmium stencils were placed around the samples to shield the scintillator from excessive neutron radiation and to investigate the influence of the backlight effect. The contribution of scattered neutrons to the total detected intensity was investigated by additionally varying the sample-detector distance and applying a functional correlation between distance and intensity. Furthermore, the influence of the surface roughness on the edge effect due to refraction was investigated.

## 1. Introduction

The degradation of mechanical properties of iron-based alloys caused by hydrogen has been a long known and well-studied problem [1]. Several mechanisms have been suggested to describe and explain the phenomenon of hydrogen embrittlement [2]. Once hydrogen uptake takes place due to welding, processing, corrosion etc., hydrogen is transported inside the sample. To understand and predict hydrogen embrittlement and hydrogen assisted cracking in iron and steel, it is important to investigate the transport and diffusion behavior of hydrogen. 

Due to the large differences in the neutron attenuation coefficients of iron and hydrogen or steel and hydrogen, respectively, neutron radiography (NR) is a suitable method to investigate the hydrogen content and distribution in steel as well as the hydrogen mass flow. The advantages of NR compared to other commonly used methods for measuring hydrogen concentrations (e.g., carrier gas hot extraction) are the non-destructiveness and the high spatial and temporal resolution [3]. Neutron radiography has already been successfully used to determine diffusion coefficients of hydrogen in metals [4], to estimate hydrogen concentration and distribution in Zircaloys [5,6] and to measure the effusion behavior of hydrogen in austenitic stainless steel [7] and technical iron [8] as function of time and temperature. The three-dimensional distribution of hydrogen in and around blisters in hydrogen-charged technical iron was investigated using neutron tomography [9].

In further experiments, we aimed for spatial- and temporal-resolved in situ NR while performing uniaxial tensile tests. Diffusion as a statistical process can be overlaid by thermodynamic driving forces in order to minimize the free energy. This allows, e.g., diffusion against the concentration gradient during precipitation build-up or spinodal decomposition. Since such driving forces can also be generated by lattice distortions [10,11], we tried to visualize such hydrogen mass flow in an elastically deformed supermartensitic and duplex stainless steel flat bar tension specimen. We expected to see the diffusion of highly attenuating hydrogen into the notched region of a tensile sample, which was held in an elastic deformation state.

However, we observed additional imaging artifacts, which could not be explained by the diffusion behavior of hydrogen in steel but rather through the interaction of neutrons with the sample and scintillator material, e.g., due to scattering or refraction effects, respectively. 

**Figure 1:**
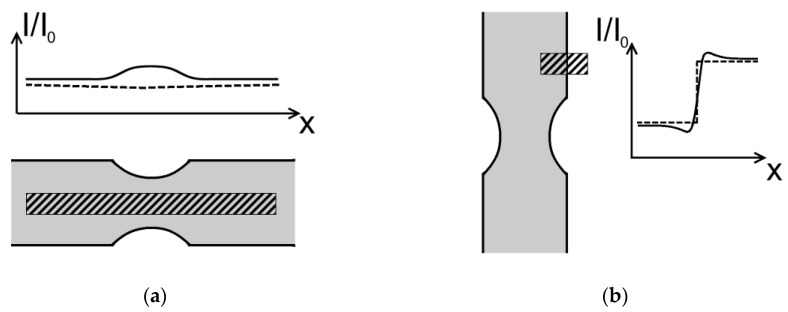
Measured (solid line) and expected (dashed line) intensity curves of regions of interest (ROI) (shaded rectangles). (**a**) ROI in a hydrogenous sample, parallel to the samples axis; (**b**) ROI in a sample without hydrogen, which covers the sample, the samples edge and air.

Figure 1a shows a sketch of the used hydrogenous tensile test sample along with the expected (dashed line) and the measured (solid line) intensity profile. A higher intensity is detected behind the sample in the notched area than in the areas on both sides of the notch. Due to the higher surface-to-volume ratio of the notched area, we expected a higher hydrogen input during electrochemical charging in the notched area than at the thicker ends of the sample. During electrochemical charging of the sample, atomic hydrogen is adsorbed at the surface and diffuses towards the inside of the sample. This leads to a hydrogen gradient in the sample with higher concentrations near the surface (~800 wt.ppm) compared to the volume (~150 wt.ppm) [12]. Since the surface is closer to the region of interest (ROI) at the notched area in Figure 1a, we expect a higher hydrogen concentration around the notched area than further away from it. This would have led to a decreased intensity level around the notched area or, if the hydrogen uptake is homogenous, to a constant intensity distribution along the ROI. 

Furthermore, we observed that hydrogen-charged samples, as well as reference samples without hydrogen, showed an intensity drop at near-surface regions (see Figure 1b), which could be interpreted as a hydrogen gradient. It is known that electrochemical charging may cause a hydrogen concentration gradient perpendicular to the surface area [13]. Yet, homogenous samples without hydrogen should not show this effect. It is thus necessary to identify and consider all contributing phenomena to correctly quantify the hydrogen distribution and concentration in our material. 

It is known that the strong scattering interaction of hydrogen with neutrons causes violations of Lambert Beer’s law and leads, e.g., to underestimated hydrogen concentrations. Scattering corrections exist through point scattered functions (PScF) for radiography [14,15,16,17,18,19] and tomography [20]. However, comprehensive knowledge of the applied neutron wavelength spectrum, detector, sample material and sample geometry is necessary. A method to correct the scattering contribution of changing hydrogen content (e.g., during in situ hydrogen charging) can be found in [21]. In general, it is recommended to increase the distance between the sample and detector to reduce the probability of detecting scattered neutrons [16]. Unfortunately, this involves a loss of the spatial resolution due to the divergence of the neutron beam. 

In general, the detected intensity I_detect_ for neutron radiography, when using an imaging set-up consisting of a scintillator, a mirror, lenses and a camera, is composed of:

- neutrons that did not interact with the irradiated material (transmitted intensity): I_trans_,
- neutrons that interacted with the material and/or experimental set-up (e.g., the mirror) through scattering and that were detected by the scintillator (scattered intensity): I_scatt_,
- neutrons that were refracted at an interface and lead to the scintillator screen: I_refract_,
- photons that were induced by (optical) scattering in the scintillator: I_backlight_ [22]. This yields the following equation:

I_detect_ = I_trans_ + I_backlight_ + I_scatt_ + I_refract_ (1)

In this study, we present investigations on the genesis of transmission images of hydrogenous steel samples using a polychromatic neutron beam. We discuss the results of systematic measurements regarding the occurrence of such image artifacts and propose general rules to reduce such error sources in quantitative image analysis.

In detail, we focused on the influence of the detector area coverage on the induced backlight signal. We investigated the contribution of scattered intensity on the scintillator subject to the sample-detector distance, and we examined the effect of the surface roughness of the sample on the occurrence of refracted neutrons on the detector.

## 2. Materials and Methods

**Figure 2:**
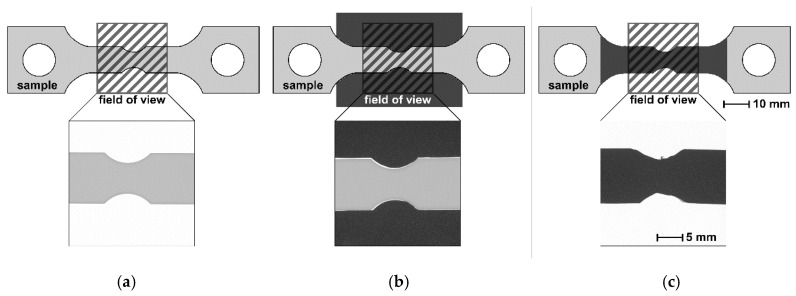
Scheme of measured flat bar tension samples and field of view of the radiographs (shaded square), which are displayed below the schemes. (**a**) Without Cd stencil, (**b**) with Cd stencil next to it (dark rectangle), (**c**) with Cd sheets (dark area) in front of sample.

Following our first observations during tensile tests, we used the same flat bar tension specimens in these experiments, shown in Figure 2, although no actual tensile test was performed. Supermartensitic stainless steel coupons were cut from a rolled sheet milled and ground to gain notched flat bar tension specimens with a thickness of 6 ± 0.10 mm. It is worth noting that the broader front of the samples was ground, in contrast to the sides, which were left with a milled surface. Using white light interferometry, we measured an average roughness for the ground surface of R_a_ = 0.69 µm and R_a_ = 5.39 µm for the milled surface. One of the samples was electrochemically charged with hydrogen in a 0.1 M H_2_SO_4_ acid solution containing 13 mg/L NaAsO_2_ (recombination inhibitor for hydrogen gas) and subsequently stored in liquid nitrogen to ensure that no hydrogen effused out of the sample before starting the measurements. The average hydrogen concentration measured at identical samples using the carrier gas hot extraction method [23] amounted to approximately 210 wt.ppm. One sample was left in the as-produced state without hydrogen charging to be used as a reference for the NR. The NR experiments are listed in Table 1.

Figure 2 shows the experimental configurations listed in Table 1.

The NR experiments listed in Table 1 were performed at the ANTARES [24] instrument operated by the Technische Universität München at the Heinz Maier-Leibnitz Zentrum (MLZ) in Garching, Germany. We used a pinhole diameter of 18 mm and a distance from pinhole-to-detector of 9 m. The exposure time for all radiographs was 80 s. The imaging set-up consisted of a Gd_2_O_2_S scintillator and a CCD camera with 13.5 µm pixel size. With the given geometry of the set-up, an effective pixel size of 12 µm could be calculated. The sample-detector distance (SDD) was used as a varying parameter (13 mm, 30 mm or 50 mm). In order to reduce backlight in the detector induced by neutrons, we placed four cadmium plates of 0.5 mm thickness (in total 2 mm) closely next to the samples like a stencil (see Figure 2b) or directly in front of the sample (see Figure 2c) for some experiments. Cadmium (Cd) has a total neutron attenuation coefficient of σ_t_ = 2470 b (at a thickness of d = 2 mm the transmission is I/I_0_ = 9.3 × 10^−11^) for a neutron energy of E = 25.3 meV (energy of flux-maximum at ANTARES instrument), whereas the total neutron attenuation coefficient of iron is σ_t_ = 14.07 b (for a thickness d = 6 mm the transmission is I/I_0_ = 0.49) [25]. The Cd plate stencil is therefore considered to be almost opaque towards the ANTARES spectrum of neutrons. The main attenuation mechanism of Cd is capturing neutrons and emitting γ radiation, which could add to the detected intensity during NR imaging. Therefore, additional measurements of intensities behind and next to the Cd plates were performed. The measured transmission behind 2 mm thick Cd accounts to I/I_0_ = 0.04. Next to the Cd sheets, no intensity addition due to emitted γ radiation was detected.

The following image correction procedures were applied for all images. First, gamma spots in the CCD detector were removed [26]. Second, dark field images and flat field images were obtained before the experiments and applied to the radiographs. In the case of in situ NR, additional regions outside of the sample projection were used to normalize the image series, avoiding intensity shifts due to beam intensity fluctuations during the acquisition. To observe and analyze the hydrogen mass flow, a carefully chosen ROI was selected inside the sample projection to avoid edge effects. Furthermore, NR was usually performed on materials of the same batch simultaneously with a second hydrogen-free sample as reference. This allowed information to be gained about absolute or relative changes.

## 3. Results and Discussion

### 3.1. Backlight Generated in Detector

**Figure 3:**
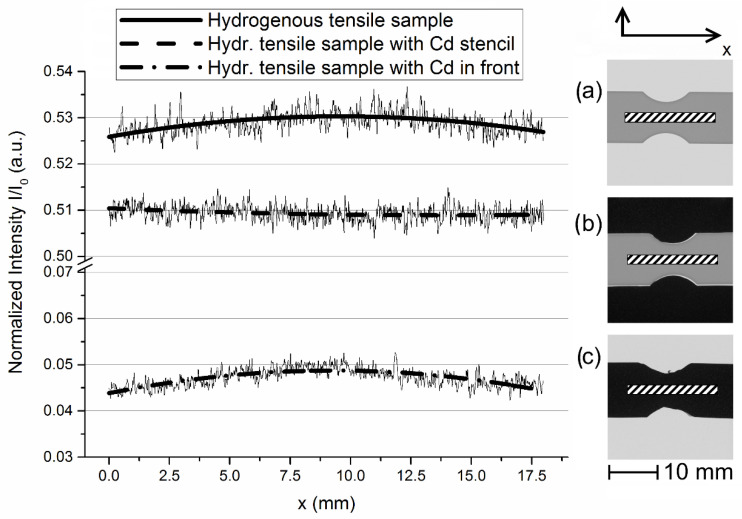
Intensity curves in ROIs shown as shaded areas in the radiographs (**a**), (**b**) and (**c**) on the right. Solid curves are Sovatzki-Golay fits (mathematical smoothing filter) of the measured intensities as a guide to the eye. (**a**) Hydrogenous tensile test sample, (**b**) hydrogenous sample framed by Cd stencil, (**c**) sample with Cd sheets in front.

Comparing radiographs taken with and without a Cd stencil next to the sample, we found a higher intensity (around 4%) behind the samples that were not surrounded by a Cd stencil (upper curve in Figure 3). Furthermore, we observed a change in the curvature of the transmitted intensity along the sample length.

We found an approximately constant intensity distribution along the sample axis in images acquired with a Cd stencil next to the sample (Figure 3b) and a maximum intensity around the notched areas for those which had no Cd stencil next to the sample (Figure 3a). Due to the electrochemical charging of the specimens, higher hydrogen concentrations are expected in the notched area. The larger surface area-to-volume ratio in this region leads to a higher hydrogen uptake during charging. Thus, lower transmission should occur in the notched area, which is not clearly visible in the sample set-up with Cd stencil. 

Although the Cd sheets in Figure 3c are expected to absorb almost all neutrons, we detected a certain intensity behind the Cd. This might be explained through the decreased cross section of Cd for epithermal neutrons, which are part of the neutron spectrum of ANTARES, and through γ-radiation, which is emitted by Cd due to interactions with neutrons. However, the intensity curve shows the same curvature and dependence from the sample shape as for Figure 3a, which hints to another source of increased intensity.

For monochromatic synchrotron radiation, detected using a Y3Al5O12:Ce scintillator and a CCD camera, it was observed that the measured intensity was overestimated when the detector screen around the sample’s projected area was not sufficiently covered from incoming radiation [22]. The authors found that the fluorescent light that is locally generated in the scintillator material by synchrotron radiation is being partially re-distributed in the scintillator due to light scattering mechanisms. The partial levelling of local intensities (i.e., overweighting small and underweighting large intensity) in one and the same sample measurement immediately suggests a partial re-distribution of the locally generated fluorescent intensity to the environment. This phenomenon is named “diffuse detector background“ or “backlight“.

Since backlight is caused by the scattering of fluorescent light generated by a scintillator, the same phenomenon should occur also for neutron imaging set-ups using a scintillator and CCD camera. This backlight could be the reason for the observed overestimation of the intensity behind the attenuating material as shown in Figure 3c with the Cd stencil. Although our ROIs in the radiographs in Figure 3 were chosen to be some millimeters away from the sample edge, the backlight influenced the quality of the measured intensity curves for the radiographs in Figure 3a,c, where the majority of the detector area was not shielded by the Cd stencil.

### 3.2. Scattering Effects

The main interactions between neutrons and atoms are absorption and scattering. As opposed to absorbed neutrons, scattered neutrons might reach the scintillator and will be detected depending on the scattering behavior of the investigated material and the distance between the sample and detector. The increase in intensity when placing the sample closer to the detector was investigated using three different source-detector distances (SDD). Next to the sample, Cd stencils where placed to reduce the contribution of the backlight effect. Due to the fact that scattered neutrons mainly do not propagate parallel to the incoming beam but are deflected by hydrogen and iron atoms, it is assumed that their contribution to the detected intensity depends on the distance between the sample and detector. The total scattering cross sections of thermal neutrons (25.3 meV) for hydrogen and iron are 82.02 b (incoherent scattering 80.26 b) and 11.62 b (incoherent scattering 0.4 b), respectively. Assuming an isotropical propagation of the incoherently scattered neutrons from a point source, the detected scattered intensity I_scatt_ is inversely proportional to the square of the distance x between scattering and detecting location:

I_total_ = I_scatt_·K/x^2^ + I_trans_ (2)

**Figure 4:**
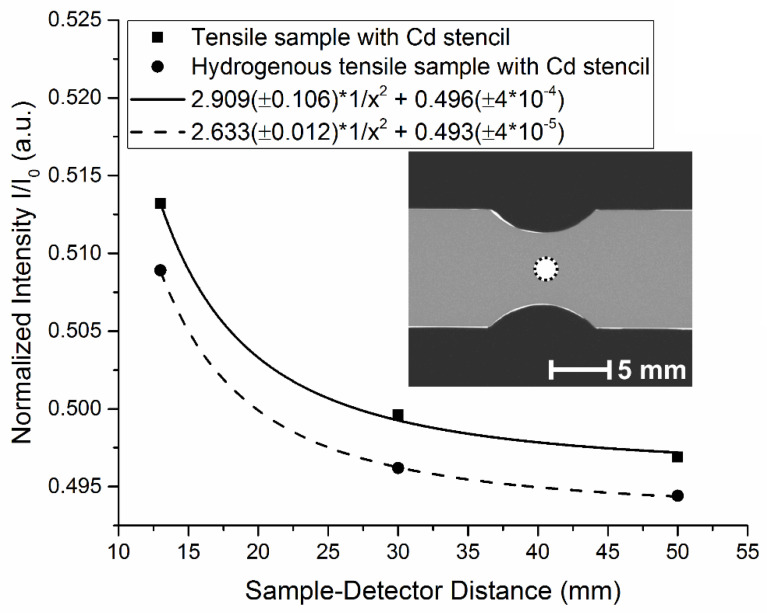
Normalized intensity for three sample-detector distances (13, 30 and 50 mm) for a flat bar tensile test specimen framed by a Cd stencil. The circle in the middle of the sample radiograph denotes the chosen ROI. The fitted curves describe an inverse-square law function of the distance.

The unknown constant of proportionality is denoted here as K. Plotting simply the average detected intensity values of ROIs in the middle of the sample (thus x ≈ SDD) as a function of SDD and fitting an inverse-square law function separates the intensity contribution I_trans_, which is generated by neutrons that pass the sample without interacting with the material and which are independent from SDD, from the total detected intensity I_total_ (see Figure 4).

### 3.3. Edge Effects Due to Scattering and Refraction

**Figure 5:**
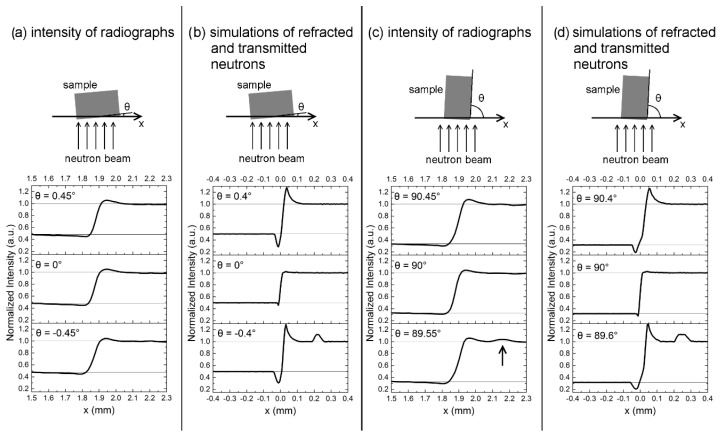
Intensity curves near a sample edge as a function of rotation angle θ. The images on top show the top view of the rotation system and the sample with a cross section of 6 × 10 mm^2^. (**a**) Measured radiograph of hydrogenous steel sample with milled surfaces aligned in parallel direction of the incoming beam. (**b**) Simulation of transmitted and refracted neutrons on a perfectly smooth surface of a simulated supermartensitic steel without additional hydrogen. (**c**) Measured radiograph of hydrogenous steel sample with ground surfaces aligned in direction of the incoming beam. The arrow in θ = 89.55° points to a second intensity maximum. (**d**) Corresponding simulation of transmitted and refracted neutrons on a perfectly smooth surface.

Another phenomenon of scattered neutrons became visible at the edges of the samples. We found an intensity gradient from the sample edge to the inside of the sample even in radiographs of steel samples, which were not charged with hydrogen. Additionally, an intensity maximum was visible just outside of the sample edges (see Figure 5a,c). This maximum is caused by neutrons that were scattered in the sample, deflected and hitting the scintillator outside of the sample’s projection [27]. Usually, this effect is visible in radiographs as a halo around the sample.

Another contributing factor to the edge effect besides scattered neutrons comes from neutrons which are refracted at the sample surfaces [28]. In Figure 5, intensity distributions of measured radiographs and simulations of absorption and refraction of neutrons with a sample-detector distance of 13 mm are shown as a function of a rotation angle θ. Figure 5a,c show the measured intensities and a top view of the experimental set-up. Figure 5b,d show the corresponding simulations. The simulations were performed with the Monte Carlo ray-tracing package McStas [29,30]. Into this package, a module was implemented that is capable of simulating absorption, refraction and total reflection in a sample with ideally flat surfaces based on microscopic properties (scattering cross section, absorption cross section, coherent scattering length, density, molar mass) of the material. From these microscopic parameters, the refraction angles at material interfaces are calculated as well as the probabilities for absorption within the material and total reflection at the surface. The instrument properties like collimation and wavelength spectrum were also considered in the simulations, whereas the spatial resolution of the detector was not considered.

The simulations show that sample sides aligned exactly parallel to the incoming beam do not lead to strong irregularities in the intensity distributions at the sample edge. Whereas, when rotating the sample, more neutrons are refracted at two non-parallel sample sides, which leads to a stronger total deflection and to intensity excess outside of the sample projection and intensity deficiency inside the sample projection on the detector. These peaks are not as clearly visible in the measured radiographs in Figure 5a,c, which might be caused by the surface roughness or limited spatial resolution of the detector. The surface roughness creates a wider distribution of angles at which the neutrons enter or leave the sample, which leads to a more random deflection of the neutrons. Another phenomenon of refraction, i.e., total reflection, is visible in the simulation when rotating the sample in a clockwise direction. An additional intensity maximum further away from the sample edge is visible in the simulations in Figure 5b for θ = 0.4° and Figure 5d for θ = 89.6°. Note that for a positive rotation angle, the intensity peak from reflected neutrons would occur on the other side of the sample. The measured intensity distribution in Figure 5c shows a small additional intensity maximum at approximately the same distance from the sample edge for a rotation angle of θ = 89.55°. This is caused by neutrons, which were reflected at the ground sample side and is, again, less pronounced than the peaks of total reflection in the simulation. Interestingly, the total reflection is not visible at all, when the rougher, milled surface is tilted towards the incoming neutron beam. In summary, the results of the experiment are in qualitative agreement with the simulations, which shows that refraction and total reflection are important aspects that need to be considered in quantitative neutron imaging experiments. The quantitative differences between simulation and experiment may be attributed to the surface roughness of the samples as well as the limited spatial resolution of the detector, which leads to a smearing of the curves. Both experimental aspects were not considered in the simulation.

## 4. Conclusions

In this work we point out some problematic effects arising when performing quantitative imaging experiments with neutrons. The intensity induced in the scintillator due to optical scattering is independent from the irradiated material and depends on the sample geometry and the distances between the ROI and highly irradiated areas of the scintillator. It can be reduced by either using the full detector area for imaging or by shielding the unused detector areas from neutrons with non-transparent material. This is recommended especially for more complex sample geometries and when intensity trends are in focus. A correction procedure for homogenous samples is given by Müller et al. [22]. 

The contribution of scattered neutrons to the detected total intensity is dependent on the sample-detector distance and the scattering behavior of the sample material. We have shown that additional measurements with varying sample-detector distances could yield the possibility to separate the transmitted intensity from the scattered intensity by fitting an appropriate distance law. Alternatively, if the loss of spatial resolution is not important, an increase of the distance between the sample and detector is a suitable approach to reduce such scattering effects. This would also reduce the shown edge effect, which is partially caused by scattered neutrons. The other contributing effect is the refraction of neutrons on smooth surfaces that are not aligned exactly parallel to the incoming beam. This shows the importance of a very accurate sample alignment. A rougher surface did not show an additional intensity peak due to total reflection.

## Figures and Tables

**Table 1:** List of neutron radiography experiments performed at the ANTARES instrument.

Sample State	Cadmium	Sample-Detector Distance (SDD)	Schematic Image in
Steel charged with H	With Cd stencil	13, 30, 50 mm	Figure 2b
	Without Cd stencil	13 mm	Figure 2a
Steel without H	With Cd stencil	13, 30, 50 mm	Figure 2b
	With Cd in front of sample	13 mm	Figure 2c
